# Hypoglossal Nerve Paraganglioma Depicting as Glomus Tumor of Neck

**DOI:** 10.22038/ijorl.2020.43602.2448

**Published:** 2021-03

**Authors:** Zaid-Ahmed Shamsi, Fareed-Ahmed Shaikh, Muhammad Wasif, Mustafa-Belal-Hafeez Chaudhry, Nadeem-Ahmed Siddiqui, Ziad Sophie

**Affiliations:** 1 *Department of Surgery, Aga Khan University Hospital, Karachi, Pakistan* *.*; 2 *Department of Surgery, Ziauddin University and Hospital.*; 3 *Department of Radiology, Shifa International Hospital, Islamabad, Pakistan.*

**Keywords:** Cyber knife, Cryo surgery, Hypoglossal nerve, Paraganglioma

## Abstract

**Introduction:**

Paraganglioma are infrequent neuroendocrine tumors that are most commonly found in the carotid body, ganglia of the vagus, jugular and tympanic nerve. Very rarely they can involve other cranial nerves outside the cranial cavity, we present one such case of hypoglossal nerve paraganglioma in neck.

**Case Report:**

A 48 years old male presented with 1-month history of right sided stroke and aphasia. Ultrasonography of neck revealed a highly vascular mass on the right side of the neck. CT angiogram confirmed a highly vascular mass arising above the carotid bifurcation. With the working diagnosis of Glomus tumor, he underwent right sided neck exploration, however, intra-operatively tumor was found to be arising from the hypoglossal nerve instead. Surgery was abandoned on basis of the available literature, with only 6 reported cases in the past 54 years. Patient had no immediate post op complications and was sent for cyber knife treatment. After completion of 5 cycles of cyber knife there was a total of 45% reduction in the size of the paraganglioma with the resolution of the patient’s symptoms after a follow up of 6 months.

**Conclusion:**

Hypoglossal nerve paraganglioma is an uncommon tumor of the neck and can be misdiagnosed with the other tumors in this region especially chemodectoma and glomus tumor. The diagnostic criteria and appropriate treatment modalities have not been established due to the rare presentation hence hypoglossal paraganliomas should be kept in mind when Highly vascular neck mass is encountered.

## Introduction

Paragangliomas are thought to arise from the neural crest cells and are usually located along with the parasympathetic nervous system. Their migration during embryonic development accounts for the unforeseen locations of Paraganglioma in the human body (1). Paragangliomas are infrequent neuroendocrine tumors that are most commonly found in the carotid body, ganglia of the vagus, jugular and tympanic nerve, the temporal bone, and within the nasal, orbital tissue or laryngeal tissue (2). Only a few case reports have been published in the past, the majority of them in the last decade, describing the different modes of presentation and treatment strategies of hypoglossal nerve paraganglioma. Pre-operative diagnosis has been reported in only one of the cases (3).

Hence, it is safe to assume that there are no established diagnostic or treatment criteria for these tumors. In this report, we describe a case of hypoglossal nerve paraganglioma with a unique presentation and its novel management.

## Case Report

A 48-year-old gentleman presented as an emergency to our institute with left-sided hemiparesis and aphasia. He had no prior co-morbidities, and the family history was insignificant. On examination, he was found to have a power of 0/5 in all muscle groups, according to the medical research council (MRC) muscle grading system, on the left side along with complete aphasia. However, the function of cranial nerve XII was intact. However, the examination was the neck was unremarkable; there was no palpable mass or skin changes in the neck region. The carotid pulses were palpable bilaterally.

**Fig 1:**
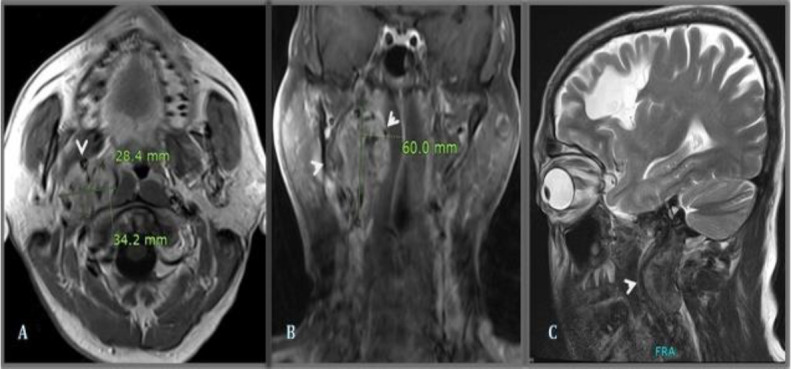
Pre-treatment MRI head and neck examination with IV gadolinium contrast. (A) T1 weighted post-contrast [axial section], (B) T1 weighted post-contrast [coronal section] and (C) T2 weighted [sagittal sections], shows an abnormal signal inten

**Fig 2:**
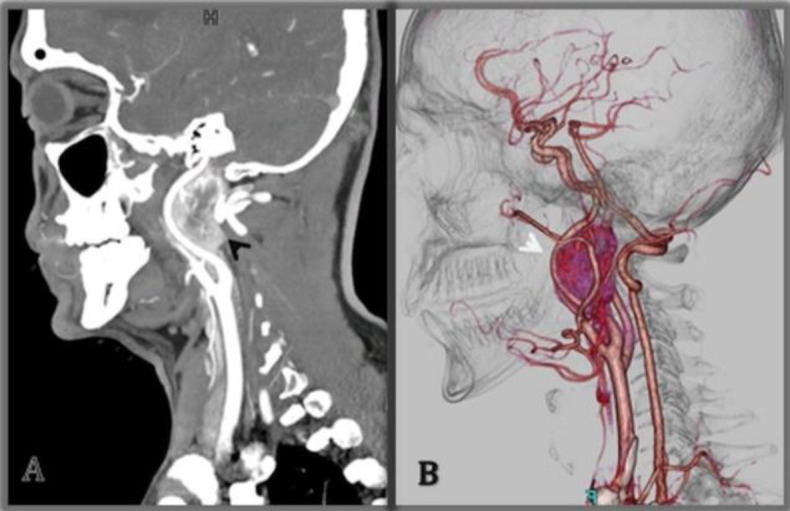
Pre-treatment CT Angiography of Carotid. (A) Post-contrast coronal section and (B) 3D reformatted image. An avidly enhancing highly vascular lesion (arrow head) behind the left internal carotid artery extending form the carotid bifurcation

On initial workup, Magnetic resonance imaging with angiogram was done, which showed a right middle cerebral artery (MCA) territory infarct and a heterogenous lesion within the neck just above the level of the carotid bifurcation. Subsequently, post-contrast images from the head and neck region were also acquired (Fig.1). The lesion's appearance and characteristics suggested a glomus tumor of the neck. Further evaluation with an ECG and cardiac echo was performed to rule out the cause of an infarct. ECG and cardiac echo were found to be normal. Computed tomography angiogram of the neck was done (Fig.2), which showed a highly vascular mass, 2.8 x 3.4 x 6.0 cm in size, arising above the carotid bifurcation with its feeding vessel arising from the right external carotid artery. It was splaying the right internal carotid artery anteriorly and medially and extending up to the hypoglossal canal.

The radiologist suggested it as an atypical glomus tumor or a rare peripheral nerve sheath tumor. With consideration of the location, clinical presentation, and imaging features, a working diagnosis of glomus tumor of the neck was made.

**Fig 3:**
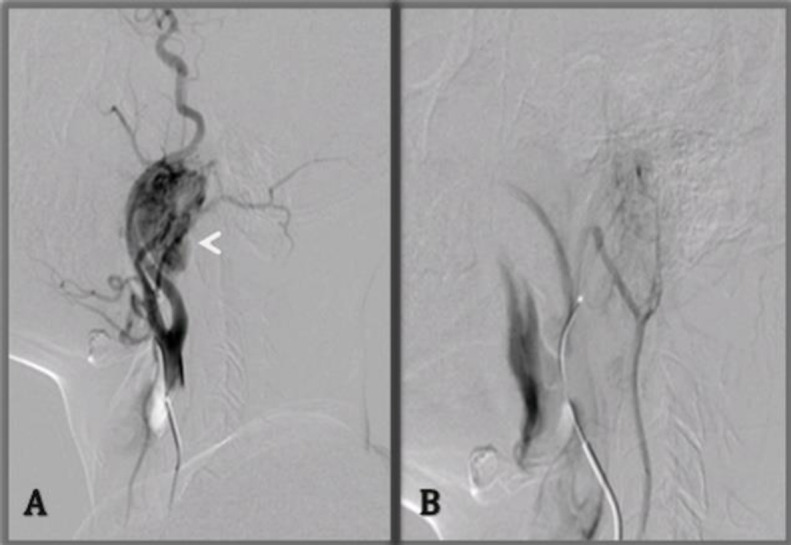
Pre-operative DSA and angioembolization of the lesion. (A) Pre-embolization-right common carotid artery angiogram and, (B) post-embolization -right external carotid arteryangiogram. Pre-embolisation run demonstrates signific

Based on our working diagnosis of Glomus tumor, surgical excision was planned. In order to control hemorrhage intra-operatively, angioembolisation of the tumor was done before surgery, and approximately 90% embolization was achieved (Fig.3).

**Fig 4:**
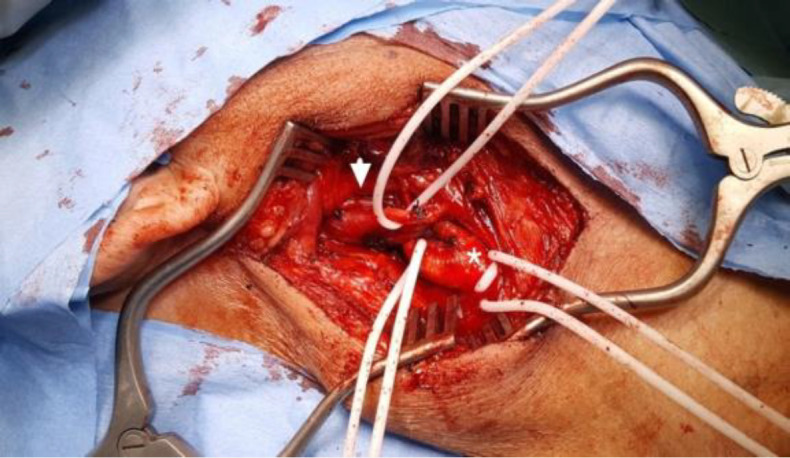
Per-operative neck dissection through anterior cervical approach. A high riding soft tissue lesion along the right hypoglossal nerve (arrow head). It is lying superior to carotid bifurcation (asterik)

Under general anesthesia, surgical excision was started using the vertical right anterior cervical approach (Fig.4). Upon exposure to the carotids, the tumor was found to be away and not compressing the internal carotid artery. 

In further dissection, it was revealed that the tumor was originating from the right hypoglossal nerve, completely encasing it, with some of its portion lying intracranially, and only the distal end was visible just below the angle of the mandible. Unsure about the further step, a literature search was done, and it was found that there were only a few cases reports regarding such tumors with at least two reports suggesting radiosurgery as an alternative to surgical management (1).

**Fig 5:**
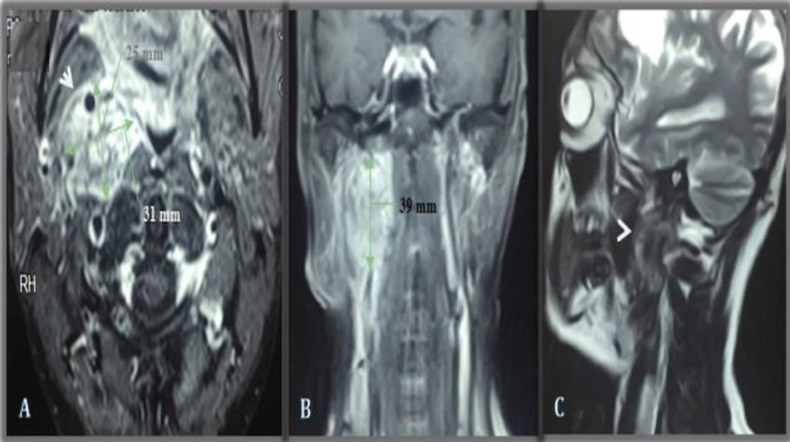
***Post-treatment MRI head and neck examination with IV gadolinium contrast. (A) T1 weighted post-contrast [axial section], (B) T1 weighted post-contrast [coronal section] and (C) T2 weighted [sagittal sections]; shows almost 45% interval dec***

 Hence, with no possibility of tumor resection without sacrificing the hypoglossal nerve, the procedure was abandoned, and the patient was planned for cyberknife therapy. No immediate post-operative complications were noted, and the patient underwent five cycles of radiosurgery (cyberknife). Three months' post-treatment MRI was done, which showed a 45% reduction in the size of the tumor (Fig.5) with significant improvement in symptoms and complete resolution of aphasia.

## Discussion

The first-ever evidence of hypoglossal Paraganglioma was described by Wilson in 1968. (1) Since then, the literature search has yielded less than ten published cases of these tumors, the majority of which were published in the last decade. The close proximity of these tumors to the carotid vessels has rendered the pre-operative diagnosis almost impossible (2). Vagal Paraganglioma displaces the ICA anteriorly and medially without the classic splaying of the carotid bifurcation (3). In this case, the tumor was found proximal to the carotid bifurcation and displaced the ICA anteriorly and medially. So far, only one case report describes the diagnosis of hypoglossal nerve paraganglioma through pre-operative imaging (2). Paraganglioma is a highly vascular tumor. They display, as in this case, a typical "salt, and pepper" appearance in T2 weighted images on MRI (4).

In our case, the tumor arising from the hypoglossal nerve was only identified per operatively, although the pre-operative imaging favored the diagnosis of a glomus tumor in the neck. Similar findings were reported by Shintani et al. (5) and Takayama et al. (6), whereby pre-operative imagining suggested an origin of tumor from the vagus nerve in the neck.

The usual presentation of hypoglossal nerve paragangliomas includes a painless neck mass with an indolent pattern of growth. This may or may not be accompanied by dysphonia, dysphagia, and altered taste. Ipsilateral tongue wasting with or without deviation may also be present (7). In our case the presentation of the patient with left-sided hemiparesis and aphasia is a unique presentation and the exact cause of the patient's symptoms could not be attributed to this mass, but the resolution of symptoms, resolution of aphasia and improvement in hemi paresis, with the decrease in the size of mass post-treatment with cyberknife shows some unknown association.

Surgical excision remains the mainstay of treatment for Paraganglioma as malignant change occurs in 12% of sporadic cases. High vascularity of these tumors has prompted surgeons towards pre-operative embolization of the tumor bed to minimize intra-operative bleeding and to aid in tumor dissection. 

However, a post-operative course after resection of hypoglossal Paraganglioma without nerve sparing has not been without complications. Complete cranial nerve XII palsy was noted by Farr et al. (2), Shintani et al. (5) and Takayama et al. (6) Tongue hemiparesis was reported by Ross et al. (8), Santovito et al. (9) and Marchesi et al. (10) Santovito and Marchesi also reported difficulty in swallowing. Ipsilateral tongue atrophy was only reported by Mehmet et al. (11), although deglutition was found to be normal in this case. Fink et al. (12), however, noted no significant complication after resection of hypoglossal Paraganglioma with the preservation of the nerve. This idea was backed by millet et al., who stated that nerve preservation, whenever possible, with excision of vagal Paraganglioma, leads to improved post-operative functionality without increasing recurrence risk (13).

Conventional fractionated external beam radiation and Gamma/cyberknife may be used as an alternative method of resection, especially in cases where tumor size is small and/or is inseparable from the nerve to reduce the morbidity related to tumor surgery (7). Gamma knife has shown to be more effective with fewer side effects when compared to conventional radiotherapy (14).

Hypoglossal Paraganglioma is an uncommon tumor of the neck and can be misdiagnosed with the other tumors in this region, especially chemodectoma and glomus tumors. The diagnostic criteria and appropriate treatment modalities have not been established due to the rare presentation; hence hypoglossal Paraganglioma should be kept in mind whenever a neck mass is encountered. Treatment strategies include complete surgical excision, if possible, without sacrificing the nerve and radiosurgery (gamma/cyberknife) for residual, irresectable, and small tumors.

## Conclusion

***Hypoglossal nerve paraganglioma is an uncommon tumor of the neck and can be misdiagnosed with the other tumors in this region especially chemodectoma and glomus tumor. The diagnostic criteria and appropriate treatment modalities have not been established due to the rare presentation hence hypoglossal paraganliomas should be kept in mind when ever a neck mass is encountered.***
